# Generalist bird exhibits site‐dependent resource selection

**DOI:** 10.1002/ece3.8016

**Published:** 2021-08-16

**Authors:** Samantha M. Cady, Craig A. Davis, Samuel D. Fuhlendorf, Rheinhardt Scholtz, Daniel R. Uden, Dirac Twidwell

**Affiliations:** ^1^ Department of Natural Resource Ecology and Management Oklahoma State University Stillwater OK USA; ^2^ Department of Agronomy and Horticulture University of Nebraska Lincoln NE USA; ^3^ School of Natural Resources University of Nebraska Lincoln NE USA

**Keywords:** birds, functional response, generalist species, resource selection, scale, wildlife management, woody cover

## Abstract

Quantifying resource selection (an organism's disproportionate use of available resources) is essential to infer habitat requirements of a species, develop management recommendations, predict species responses to changing conditions, and improve our understanding of the processes that underlie ecological patterns. Because study sites, even within the same region, can differ in both the amount and the arrangement of cover types, our objective was to determine whether proximal sites can yield markedly different resource selection results for a generalist bird, northern bobwhite (*Colinus virginianus*). We used 5 years of telemetry locations and newly developed land cover data at two, geographically distinct but relatively close sites in the south‐central semi‐arid prairies of North America. We fit a series of generalized linear mixed models and used an information‐theoretic model comparison approach to identify and compare resource selection patterns at each site. We determined that the importance of different cover types to northern bobwhite is site‐dependent on relatively similar and nearby sites. Specifically, whether bobwhite selected for shrub cover and whether they strongly avoided trees, depended on the study site in focus. Additionally, the spatial scale of selection was nearly an order of magnitude different between the cover types. Our study demonstrates that—even for one of the most intensively studied species in the world—we may oversimplify resource selection by using a single study site approach. Managing the trade‐offs between practical, generalized conclusions and precise but complex conclusions is one of the central challenges in applied ecology. However, we caution against setting recommendations for broad extents based on information gathered at small extents, even for a generalist species at adjacent sites. Before extrapolating information to areas beyond the data collected, managers should account for local differences in the availability, arrangement, and scaling of resources.

## Graphical abstract

Though it is known that animals can show conditional selection responses (e.g., differential habitat selection in different areas of their range, under variable weather conditions, or in response to variable food availability), we test a scenario where ecologists might reasonably assume habitat selection patterns to be static. Specifically, we found marked differences in the direction, magnitude, and scale of habitat selection by a generalist bird at two, proximal study sites in the same area of the species' range. Our study demonstrates that—even for one of the most intensively studied species in the world—we are likely to oversimplify habitat selection by using a single study site approach.

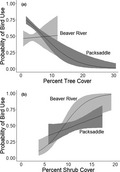

## 1 INTRODUCTION

Quantifying resource selection—an organism's disproportionate use of available resources (Johnson, 1980)—is essential for applied ecologists to infer habitat requirements of a species. For example, an organism's biological requirements can be altered by multiple processes such as thermal variability (e.g., Carroll et al., 2015), food availability (e.g., Dupke et al., 2017; Gittleman & Harvey, 1982), perceived predation risk (e.g., Lagos et al., 1995), and population density (e.g., Benson et al., 2006), leading to spatial and temporal shifts in resource selection. Developing a comprehensive picture of a species' resource requirements allows researchers to create management recommendations based on those needs. However, because resource selection is typically quantified by comparing use versus available resources, any conclusions drawn are highly conditional on the resources available to the study organism at the time and location of data collection (Beyer et al., 2010; Mysterud & Ims, 1998) and our ability to accurately describe them.

Delineating resource availability is challenging and always somewhat subjective (Beyer et al., 2010), as decisions must be made regarding the scale of availability (e.g., deciding whether areas considered available to an organism are within or outside the individual's home range) and which of these areas are actually accessible to the species. Notably, how a study defines availability can influence resource selection simply by nature of its derivation because the decision directly influences the denominator in a (% use)/(% availability) resource selection function (the class of model generally used to understand an organism or population's resource selection patterns; Manly et al., 2002). This built‐in arbitrariness of resource selection functions may lead to erroneous conclusions if availability is delineated inappropriately for the organism or research objective. An added challenge arises because landscapes are, by definition, spatially heterogeneous and patchy (Turner, 1989). Within a species' distribution, the location of a study site (in this case, referring to the location of data collection within the context of a species' range) can determine the amount, quality, and configuration of land cover types available to the organism. Studies explicitly examining the influence of site on resource selection results have largely found evidence of site‐dependent selection trends (Mcnew et al., 2013; Shirk et al., 2014; Wan et al., 2017). However, less is known about the influence of close and relatively similar sites on the resource selection patterns of generalist species.

**FIGURE 1:**
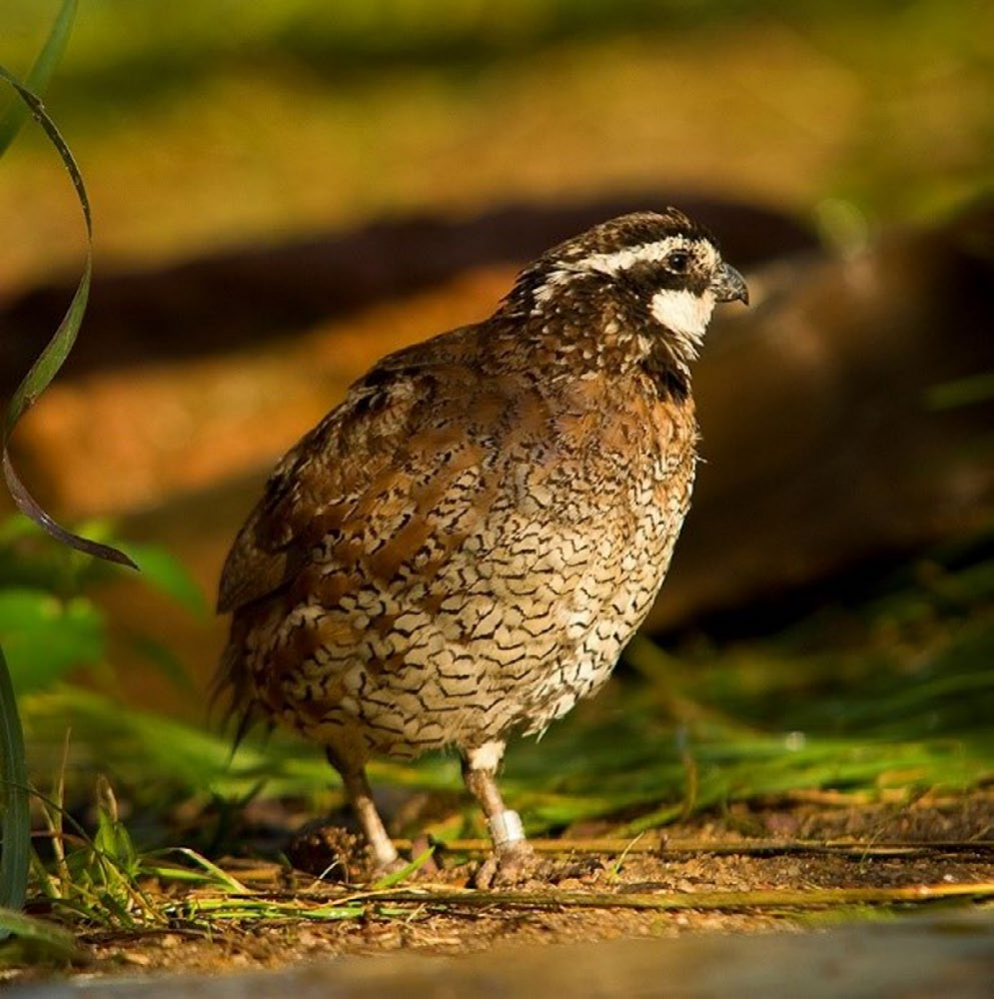
Northern bobwhite (*Colinus virginianus*). Photo credit: Todd Johnson, Oklahoma Cooperative Extension Service

As the Anthropocene continues (Crutzen, 2006), ecologists anticipate a disproportionate representation of generalist species (i.e., species that are widespread and broadly adapted; Mckinney & Lockwood, 1999), a trend which has already been documented in some communities (e.g., Davey et al., 2012; Viol et al., 2012). As a result, it will become increasingly important for managers to understand the habitat needs of generalist species, which can be complex. For example, generalist species have shown differential resource selection patterns in response to variable habitat composition (Roever et al., 2012), food availability (Hansen et al., 2009), and weather conditions (Sunde et al., 2014). Here, we further investigate this pattern by determining whether generalist species may also exhibit functional responses at similar, nearby, study sites. We selected northern bobwhite (*Colinus virginianus,* hereafter “bobwhite”; Figure 1), an intensively studied, generalist bird, as a model organism because we already have a strong understanding of their basic habitat requirements. We therefore can select variables already known to influence this species, in this case woody cover (Carroll et al., 2015). Additionally, as a nonmigratory species, we have added confidence that environmental variables occurring in regions other than our study areas will not influence the bird and confound results.

It is now widely understood that spatial scale is inherently related to space use (Johnson, 1980; Mayor et al., 2009; Whittingham et al., 2005)—that is, selection decisions are not necessarily preserved across multiple spatial scales (Mayor et al., 2009). For precision, we note although the term “scale” can refer to spatial, temporal, or organizational grains (unit of resolution) or extents (study area boundary), we use it here as shorthand for spatial grain. In recent decades, there has been increased effort to identify the “proper” scale of resource selection from a species‐specific perspective (McGarigal et al., 2016). However, any identified scale of wildlife resource selection can plausibly be different between two landscapes, even if they are nearby—yet, little is known about the influence of proximal study site location on the scaling of resource selection.

A challenge with developing a comprehensive science‐based approach to large‐scale resource selection is that replicating large landscapes is logistically difficult, expensive, and time‐intensive. Broadly, we aim to contribute to a more comprehensive model of wildlife resource selection by examining the selection patterns of a common and well‐studied, generalist species. Specifically, we use two, nearby study sites (140 km apart, which is arguably close in the context of the species' entire range) and 5 years of bobwhite movement data to determine whether proximal sites have the capacity to yield markedly different resource selection results for a generalist species. Additionally, because wildlife selects different habitat types at different scales (Anderson et al., 2005; Beatty et al., 2014; Mayor et al., 2009), we investigate whether the scale(s) at which species select their habitat is divergent between sites. Finally, we compare the differences in potential habitat availability at randomly selected landscapes with actual quail resource selection patterns.

## 2 MATERIALS AND METHODS

### 2.1 Resource selection analysis

#### 2.1.1 Study sites

**FIGURE 2:**
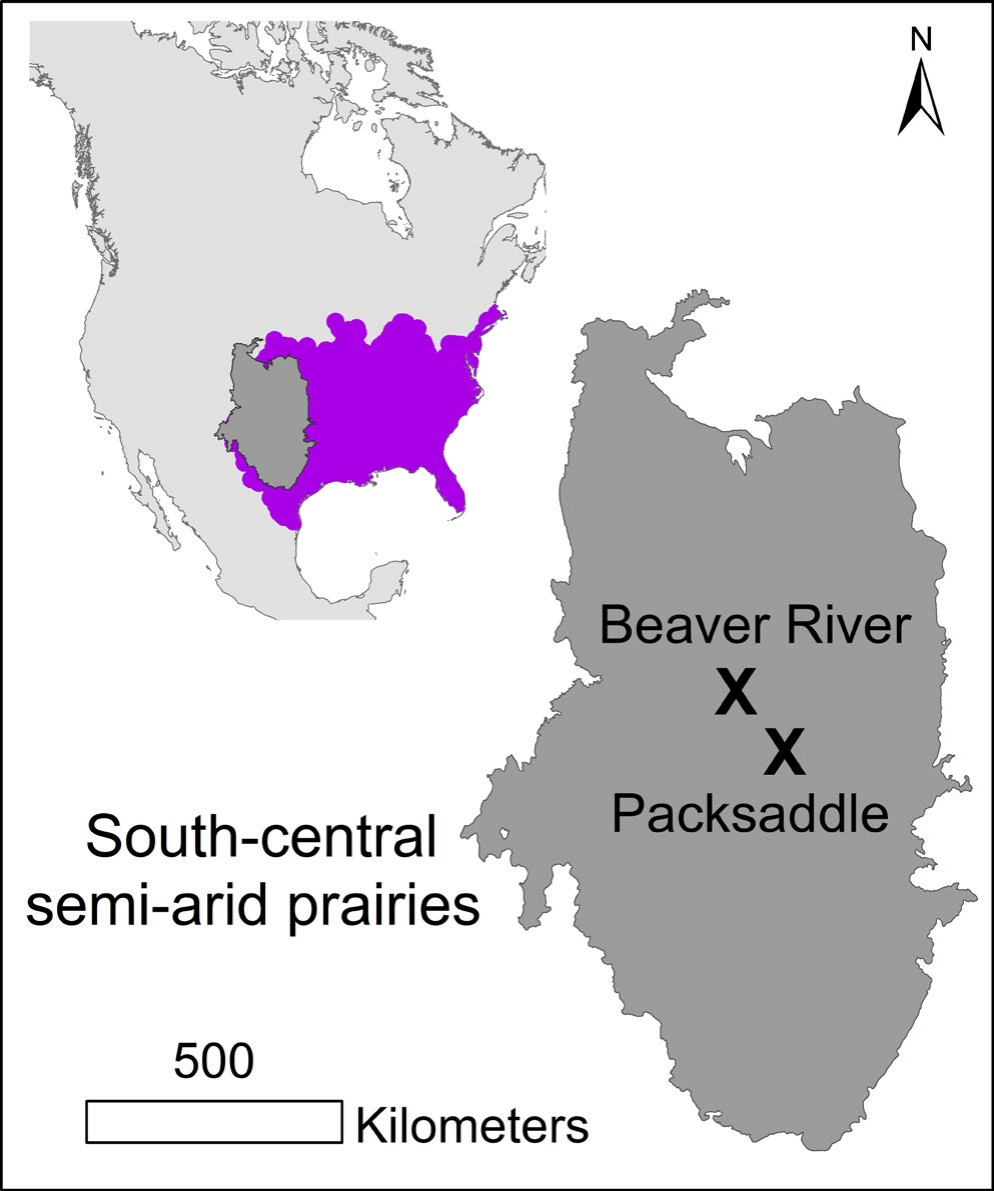
Beaver River and Packsaddle WMA in the south‐central semi‐arid prairies of North America. The purple polygon represents the northern bobwhite's range and was compiled using the North American Breeding Bird Survey data from 1967 to 2018 (only includes bobwhite in the contiguous United States; Pardieck et al., 2020)

This study was conducted in the south‐central semi‐arid prairies of North America, on two Oklahoma wildlife management areas (“WMA”; Figure 2) managed by the Oklahoma Department of Wildlife Conservation, mostly for hunting and cattle grazing. Both WMAs are located on the western margin of the bobwhite's range and are approximately 140 km apart. A distance of 140 km is arguably proximal within the context of the entire species' continental range, which extends to the east coast of North America and includes diverse ecoregions (Figure 2.). Beaver River WMA (“Beaver River,” WGS 36.8293, −100.664) includes approximately 7,200 ha of southwestern tablelands and high plains, dominated by sandsage grassland and shortgrass prairie (Tyrl et al., 2008). Packsaddle WMA (“Packsaddle,” WGS 35.883, −99.6591) is 8,100 ha of central Great Plains, where the dominant vegetation includes mixed grass prairie with shinnery oak grassland (Tyrl et al., 2008).

Vegetation composition and configuration differ between the two sites. Specifically, woody vegetation on Packsaddle is comprised mostly of shinnery oak (*Quercus harvardii*), but also includes sand sagebrush (*Artemisia filifolia*) and sand plum (*Prunus angustifolia;* DeMaso et al., 1997). Tall woody vegetation at Packsaddle is mostly hybrid sand shinnery/post oak (*Quercus havardii* × *Quercus stellata)* and occasionally cottonwood (*Populus deltoides*), hackberry (*Celtis occidentalis*), soapberry (*Sapindus drummondii*), and black locust (*Robinia pseudoacacia*; Rakowski et al., 2019), whereas at Beaver River, woody cover is dominated by sand sagebrush (*Artemisia filifolia*) with occasional sand plum (*Prunus angustifolia*) in the uplands, along with salt cedar (*Tamarix* spp), hackberry (*Celtis occidentalis*), American elm (*Ulmus americana*), and sand plum (*Prunus angustifolia*) in the floodplains and river bottom (Atuo & O’Connell, 2017). Packsaddle and Beaver River have both been subjected to oil and gas development, though well activity is more active and extensive at Packsaddle. Additionally, both sites are managed using cattle grazing and prescribed fire, but Packsaddle is burned much more frequently than Beaver River.

#### 2.1.2 Bird location data

Bobwhite movement data were collected from wild birds on both WMAs from 2012 to 2016. Adult bobwhite were captured using walk‐in funnel traps, fitted with a VHF radiocollar, and located using radiotelemetry approximately 4–7 times per week. Trapping effort was high; 2,399 trap locations were established at Packsaddle, and 1,382 were established at Beaver River. It is worth noting that trap effort was not uniform across the study sites (Appendix 1) and trapping intensity tended to be higher along roads. Though some bobwhite individuals were tracked year‐round, to mitigate confounding factors (e.g., uneven seasonal sampling between sites) and improve inference confidence, we limited analysis to bird locations collected during the breeding season (April–September; e.g., Carroll et al., 2017; Carroll et al., 2017). To increase sample independence, duplicated locations were removed by (a) including only one bird per covey and (b) including only one point at a nest location. If a bird location did not occur on a pixel with land cover data (i.e., not a rangeland pixel and not associated with Rangeland Analysis Platform data), it was not included in analysis. During the 5‐year study period, 35,499 locations were recorded from 1,725 birds across both sites and used in analysis (21,172 locations from 968 birds on Packsaddle and 14,327 locations from 757 birds on Beaver River). Each telemetry fix (bird GPS location) was considered a “presence” to be compared with “pseudo‐absences” (described in more detail in the Statistical Analysis section). A more comprehensive description of the dataset and field methods is detailed in Davis et al. (2017).

#### 2.1.3 Environmental variables

Our research objectives require high‐resolution, continuous environmental data, and the Rangeland Analysis Platform (Jones et al., 2018) is well‐suited to meet these needs. The raster dataset contains annual‐scale, continuous percent land cover data for multiple plant functional groups at approximately 30‐m resolution and is freely available online (https://rangelands.app/). The percent cover data were generated by compiling field‐collected data from approximately 60,000 field plots along with over 200 layers of gridded surface data and a random forest model to predict functional cover types across the western half of the United States (Jones et al., 2018). The predictive accuracy of the Rangeland Analysis Platform (Cover version 1.0) included 6.9% mean absolute error for the shrub layer and 4.7% for the tree layer. Because our objectives are to identify broad selection trends at medium to large scales (0.81–1,739 ha), and because we are not investigating thresholds or change over time, we are confident that the Rangeland Analysis Platform is appropriate for our purposes. Because it is well‐known that woody cover is important for bobwhite (e.g., Carroll et al., 2015), we included both shrub cover and tree cover functional groups in analysis.

#### 2.1.4 Statistical analysis

We excluded all nonrangeland pixels in the WMAs (e.g., standing water, agriculture, roads) because the Rangeland Analysis Platform algorithm is designed to best predict rangeland cover types. To simultaneously test bobwhite responses to shrub and tree cover at multiple scales, we systematically scaled up both cover classes (i.e., averaged pixel neighborhoods by moving windows). Moving window sizes (of 30‐m resolution pixels) included 3 × 3, 9 × 9, 27 × 27, 81 × 81, 113 × 113, and 139 × 139. This resulted in 30‐m resolution percent cover data that were aggregated to incorporate 0.81 ha (90 m × 90 m), 7.29 ha (270 m × 270 m), 65.61 ha (810 m × 810 m), 590 ha (2.43 km × 2.43 km), 1,149 ha (3.39 km × 3.39 km), and 1,739 ha (4.17 km × 4.17 km) of surrounding landscape context (i.e., the grain resolution remained 30 m, but included average percent cover at various sized moving windows). In other words, the spatial resolution was preserved at 30 m at all spatial scales because we used a moving window rather than scaling up the raster to a lower resolution. We intentionally selected a wide range of scales, encompassing several orders of magnitude (less than 1 ha up to 1,739 ha), to allow bobwhite use to determine the appropriate scale of selection (using the telemetry data and model ranking, explained in more detail below). Percent cover data for all moving window sizes (each size to represent a spatial scale) and cover types were extracted to each bird location in each year (i.e., the telemetry year was matched with the year of the land cover data). For example, a bobwhite telemetry location collected in 2012 would have 12 environmental variables associated with it, including 6 scales of shrub cover and 6 scales of tree cover.

We generated random‐point pseudo‐absences in each given year and in equal proportion to presence data (i.e., one absence point per presence point) within each study region (Packsaddle and Beaver River, including a 500 m buffer around the WMA boundary to include birds that were tracked slightly outside the WMA boundary lines) to function as unused habitat in the models. In other words, 35,499 bobwhite presence locations—each paired with a randomly generated absence location—resulted in a dataset of 70,998 presence/pseudo‐absence data points. It is important to note that mitigating trap bias while delineating available, unused habitat is an inherent challenge in space use/resource selection analyses (Millspaugh & Marzluff, 2001). Because our objective was to investigate large‐scale resource selection patterns at a population level (comparable to a second‐order approach; Johnson, 1980), we defined “available” habitat as the entire buffered WMAs. At Beaver River and Packsaddle, the average long‐distance movement of bobwhite (>1000 m) was approximately 2,364 m and 2,940 m, respectively. Because 100.0% of Packsaddle and 99.5% of Beaver River were less than the average long‐distance movement from a known bobwhite location, tagged birds could have reasonably dispersed almost anywhere on the buffered WMA. Therefore, trapping intensity and the number of birds tracked were high enough to justify using the entire study area as available habitat.

All parameters were estimated, and model comparison was conducted using R v3.6.2 (R Core Team, 2020). For both of the land cover classes (percent cover of trees and shrubs), we created a series of generalized linear mixed models, where bird location/absence was the binary response variable, modeled as a function of percent cover at each spatial scale (0.81 ha, 7.29 ha, 65.61 ha, 590 ha, 1,149 ha, and 1,739 ha) using a binomial error distribution and logit link function in R package “lme4” (Bates et al., 2015). Year was included as a random slope in all models to adjust for variance attributable to yearly differences in bobwhite habitat selection (e.g., birds more likely to use woody cover in hot years). To determine whether site influences scale of resource selection, Beaver River and Packsaddle were modeled separately. We assessed the models using two approaches in order to explore two different facets of resource selection. First, we assessed the overall most important woody cover type for bobwhite at each site using Akaike information criterion (AIC) (i.e., both cover types at all scales ranked in the same AIC), using R package “bbmle” (Bolker & R Core Team, 2017). Second, in order to determine the scale of bobwhite resource selection of each environmental variable, we ranked the models using AIC for each site and environmental variable (shrubs and trees at both sites, each ranked in separate AICs). For all models, 95% confidence intervals were estimated via bootstrapping using 1,000 iterations in R package “lme4” (Bates et al., 2015). Models with delta AIC <2.0 were considered competitive, unless a null model was also competitive or if bootstrapped confidence intervals overlapped zero.

### 2.2 Randomly selected site simulations

Within the south‐central semi‐arid prairies ecoregion in North America, and using the Rangeland Analysis Platform, we generated 100, randomly located, 10 km × 10 km, landscapes for each of the three cover types (trees, shrubs, and bare ground) and compared resource availability at each landscape to known bobwhite resource selection. We calculated the mean tree cover, shrub cover, and bare ground used by bobwhite on one site (Packsaddle WMA) and compared it to the percent cover available at each simulated landscape in order to determine whether the location of a site determines whether tree cover availability is lower or higher than average use. We also examined the differences in scaling of each environmental variable across the randomly selected landscapes by varying the resolution of each landscape (systematically scaling up each landscape 100 times, while holding the extent at a constant 10 km × 10 km). The finest resolution was the original RAP data (30 m resolution); the coarsest was 3,000 m by 3000 m. At each resolution, we calculated the overall mean percent cover and the between‐cell variance of each cover type.

## 3 RESULTS

Packsaddle had a higher mean density of tree cover than Beaver River, but the WMAs were comparable in terms of average shrub cover (Table 1). For both tree cover and shrub cover, measurements were highly correlated across spatial scales (Appendix 2).

**TABLE 1:** Mean and standard deviation of percent land cover per 30 m pixel on Packsaddle and Beaver River WMA from 2012–2016

	Packsaddle	Beaver river
Tree cover	9.7 ± 9.2%	3.3 ± 3.9%
Shrub cover	10.4 ± 4.3%	9.7 ± 3.5%

### 3.1 Wildlife resource selection

**FIGURE 3:**
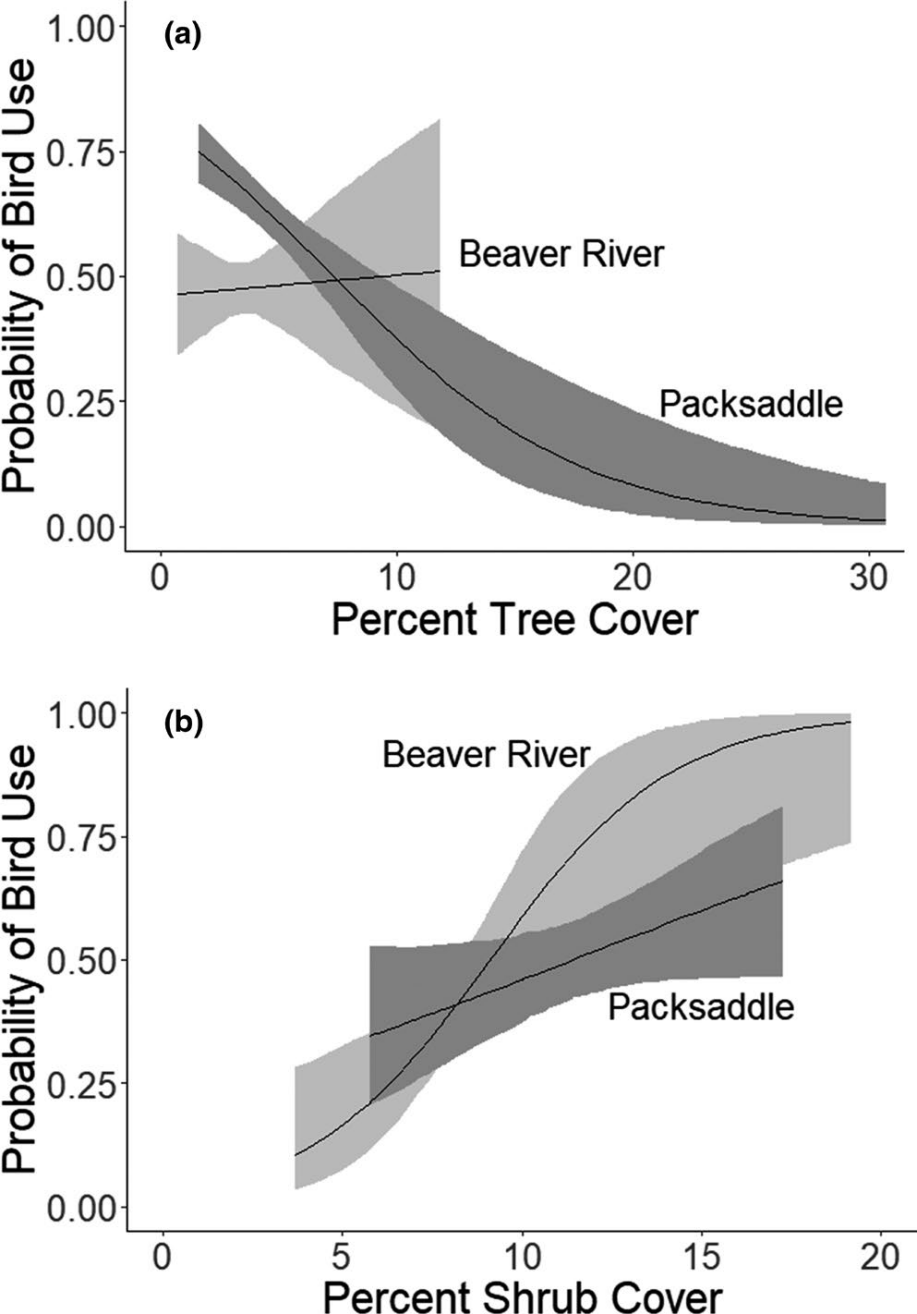
Probability of bobwhite resource selection at Packsaddle and Beaver River WMA as a function of (a) percent tree cover and (b) percent shrub cover. The spatial scale used for each estimation was selected from the top‐performing model according to AIC

**FIGURE 4:**
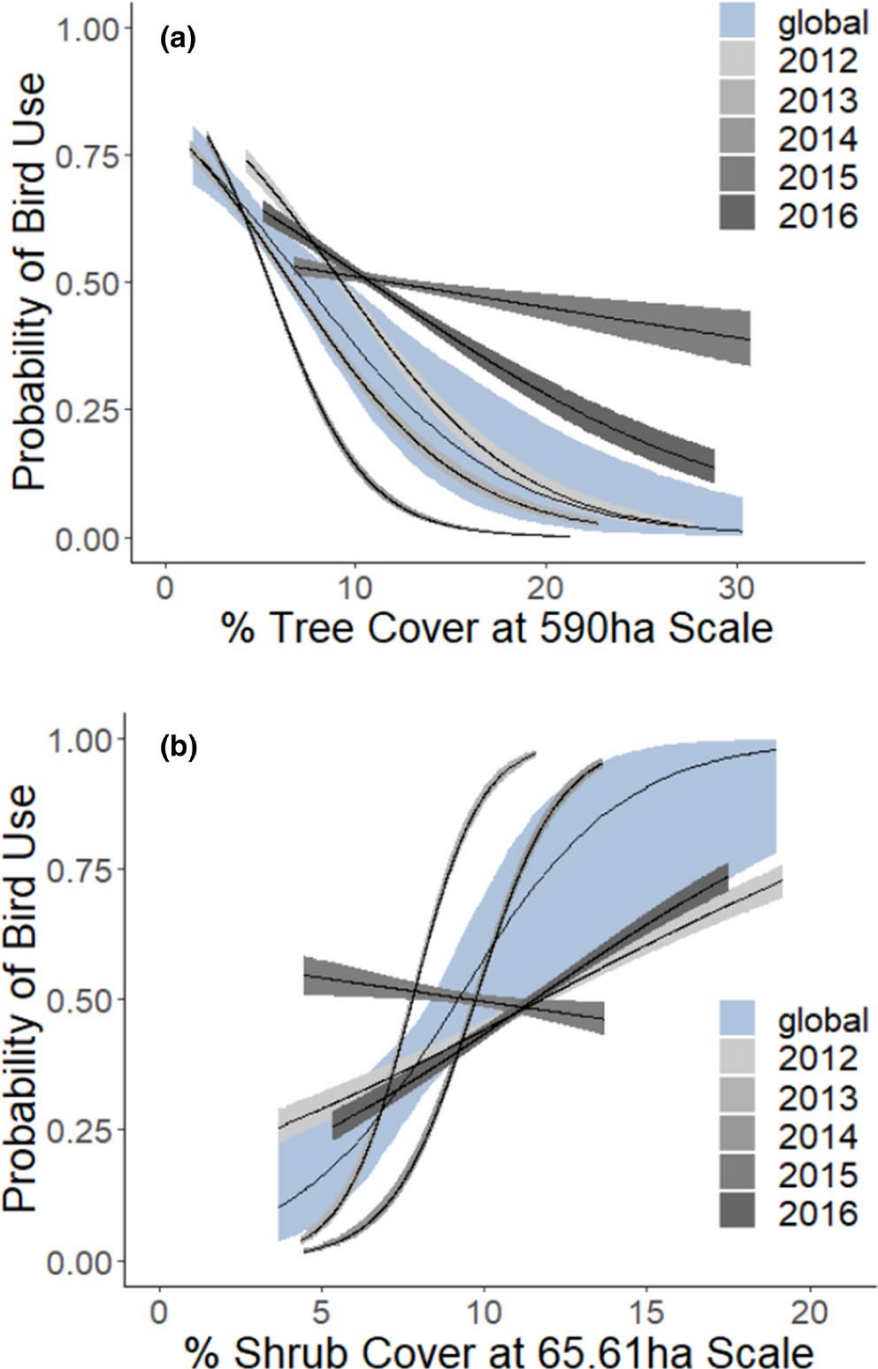
Probability of bobwhite resource selection (by year and overall) as a function of (a) percent tree cover at Packsaddle WMA and (b) percent shrub cover at Beaver River WMA. The spatial scale used for each estimation was selected from the top‐performing model according to AIC. This figure is to illustrate yearly variation (the spread of random effect groups)—all other inference in this paper refers to the global (averaged) model (blue)

According to AIC model ranks, bobwhite resource selection varied by study site. That is, we found differences in both the relative importance of cover types across the two sites. The top‐performing model for resource selection at Packsaddle WMA included a negative relationship with tree cover (*β* = −0.19), whereas the top model for Beaver River WMA indicated a positive association with shrub cover (*β* = +0.40; Table 2). Although bobwhite responded strongly and negatively to tree cover at Packsaddle (i.e., bobwhite habitat use was less likely in areas with high tree cover), we found no response to tree cover at Beaver River (Table 3, Figure 3). Conversely, though bobwhite responded strongly and positively to shrub cover at Beaver River, we found no bobwhite response to shrub cover at Packsaddle (Table 3, Figure 3). There was considerable between‐year variation in shrub selection at Beaver River and tree selection at Packsaddle WMA (Figure 4). The comparative direction and strength of effects were similar across all spatial scales, except selection against tree cover was similar between the two sites at small scales and selection for shrub cover was similar between sites at large spatial scales (Appendix 3). There was also variability among the scale of resource selection between environmental variables. Specifically, at Beaver River bobwhite perceived shrub cover at a considerably smaller spatial scale (65.61 ha) than they perceived tree cover at Packsaddle (590 ha).

**TABLE 2:** Northern bobwhite resource selection by site

	Cover type	Spatial scale	ΔAIC	Weight	*β*	95% CI
Packsaddle WMA	**Tree** Tree Tree Tree Tree Tree Shrub Shrub Shrub Shrub Shrub Shrub Null	**590 ha** 1,149 ha 1,739 ha 65.61 ha 7.29 ha 0.81 ha 1,739 ha 1,149 ha 590 ha 0.81 ha 7.29 ha 65.61 ha NA	**0.0** 307.4 307.7 851.1 2074.4 2,766.8 2,949.3 3,086.9 3,306.0 3,451.1 3,465.8 3,489.2 3,538.2	**1** <0.001 <0.001 <0.001 <0.001 <0.001 <0.001 <0.001 <0.001 <0.001 <0.001 <0.001 <0.001	**−0.19** −0.20 −0.21 −0.13 −0.07 −0.04 +0.12 +0.10 +0.05 +0.03 +0.01 +0.01 NA	**−0.31, –0.08** −0.31, −0.08 −0.32, −0.10 −0.21, −0.05 −0.12, −0.03 −0.07, −0.02 −0.06, +0.29 −0.05, +0.25 −0.04, +0.13 +0.01, +0.05 −0.00, +0.05 −0.03, +0.05 NA
Beaver River WMA	**Shrub** Shrub Shrub Shrub Shrub Tree Shrub Tree Tree Tree Tree Tree Null	**65.61 ha** 590 ha 7.29 ha 0.81 ha 1,149 ha 590 ha 1,739 ha 1,739 ha 1,149 ha 65.61 ha 7.29 ha 0.81 ha NA	**0.0** 372.8 556.1 826.6 908.3 1,309.1 1,354.5 1,444.0 1,457.7 1698.2 1999.2 2,143.6 2,324.8	**1** <0.001 <0.001 <0.001 <0.001 <0.001 <0.001 <0.001 <0.001 <0.001 <0.001 <0.001 <0.001	**+0.40** +0.45 +0.28 +0.21 +0.42 +0.01 +0.38 +0.12 +0.06 −0.02 −0.03 −0.01 NA	**+0.05, +0.74** +0.02, +0.86 +0.06, +0.51 +0.05, +0.38 +0.01, +0.85 −0.22, +0.25 −0.03, +0.79 −0.13, +0.38 −0.18, +0.31 −0.16, +0.12 −0.10, +0.05 −0.06, +0.04 NA

Note

Models with delta AIC <2.0 were considered competitive, unless a null model was also competitive or if 95% bootstrapped confidence intervals overlapped zero.

**TABLE 3:** Spatial scale of bobwhite resource selection by environmental variable and study site

	Spatial scale	ΔAIC	Weight	*β*	95% CI
*Packsaddle*					
Tree cover	**590 ha** 1,149 ha 1,739 ha 65.61 ha 7.29 ha 0.81 ha Null	**0.0** 307.4 307.7 851.1 2074.4 2,766.8 3,538.9	**1** <0.001 <0.001 <0.001 <0.001 <0.001 <0.001	**−0.19** −0.20 −0.21 −0.13 −0.07 −0.04 NA	**−0.31, −0.08** −0.31, −0.08 −0.32, −0.10 −0.21, −0.05 −0.12, −0.03 −0.07, −0.02 NA
Shrub cover	1,739 ha 1,149 ha 590 ha 0.81 ha 7.29 ha 65.61 ha NULL	0.0 137.6 356.7 501.8 516.5 539.9 589.6	1 <0.001 <0.001 <0.001 <0.001 <0.001 <0.001	+0.12 +0.10 +0.05 +0.03 +0.01 +0.01 NA	−0.06, +0.29 −0.05, +0.25 −0.04, +0.13 +0.01, +0.05 −0.00, +0.05 −0.03, +0.05 NA
*Beaver river*					
Tree cover	590 ha 1,739 ha 1,149 ha 65.61 ha 7.29 ha 0.81 ha NULL	0.0 134.9 148.6 389.1 690.4 834.4 1,015.7	1 <0.001 <0.001 <0.001 <0.001 <0.001 <0.001	+0.01 +0.12 +0.06 −0.02 −0.03 −0.01 NA	−0.22, +0.25 −0.13, +0.38 −0.18, +0.31 −0.16, +0.12 −0.10, +0.05 −0.06, +0.04 NA
Shrub cover	**65.61 ha** 590 ha 7.29 ha 0.81 ha 1,149 ha 1,739 ha NULL	**0.0** 372.8 556.1 826.6 908.3 1,354.5 2,324.8	**1** <0.001 <0.001 <0.001 <0.001 <0.001 <0.001	**+0.40** +0.45 +0.28 +0.21 +0.42 +0.38 NA	**+0.05, +0.74** +0.02, +0.86 +0.06, +0.51 +0.05, +0.38 +0.01, +0.85 −0.03, +0.79 NA

Note

Models with delta AIC <2.0 were considered competitive, unless a null model was also competitive or if 95% bootstrapped confidence intervals overlapped zero.

### 3.2 Randomly selected landscape comparisons

**FIGURE 5:**
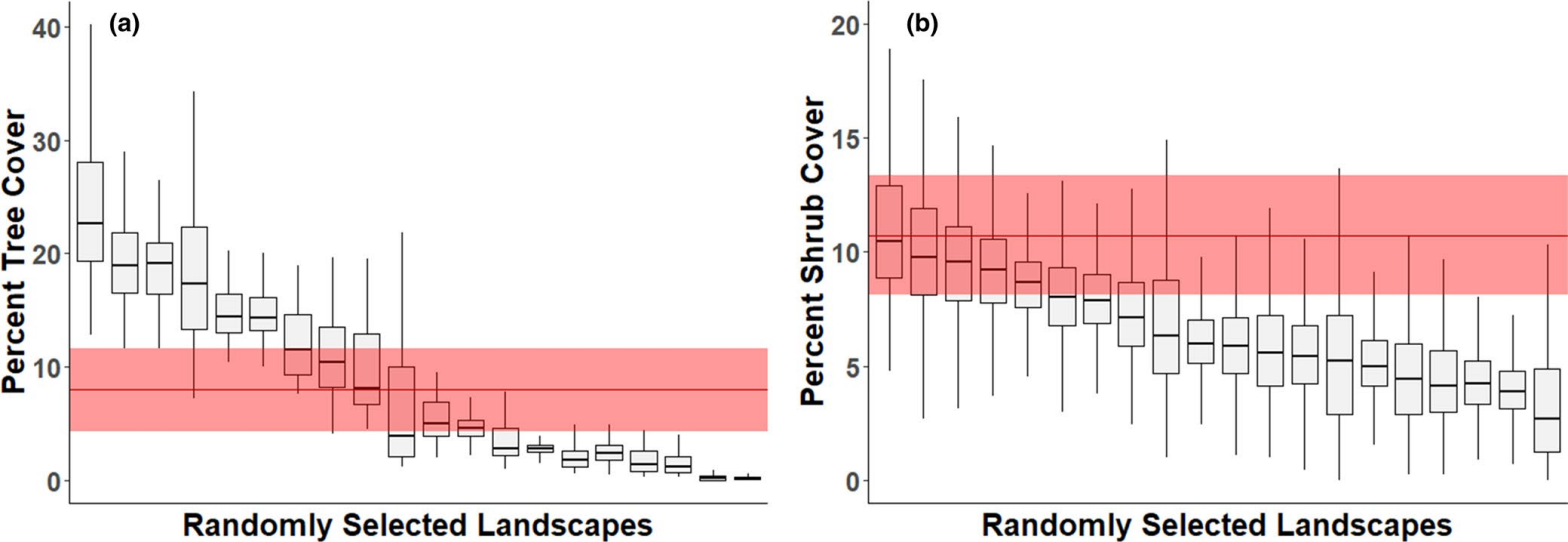
Percent woody cover composition of 100 randomly selected, 10 km × 10 km landscapes in the south‐central semi‐arid prairies of North America compared with actual bobwhite resource selection. The red ribbon represents mean percent cover (±1 standard deviation) actually used by bobwhite on (a) Packsaddle (trees) or (b) Beaver River (shrubs)

Across 100, randomly sampled, 10 km by 10 km landscapes in the south‐central semi‐arid prairies, mean percent cover of each of 6 cover classes ranged widely, yet remained relatively constant across spatial scales (Appendix 4). Further, we found that, although the general trend was between‐pixel variance decreasing with increasing scale, the magnitude (slope) varied across landscapes (Appendix 4), indicating substantial scaling differences across landscapes in the same ecoregion. Moreover, the location of a study area determines whether the average percent cover of both cover types available to the bird is within or outside of average bobwhite resource selection (Figure 5).

## 4 DISCUSSION

Resource selection methods can be applied for many reasons, which include identifying management recommendations that promote optimal habitat (Chandler & King, 2011; Suárez‐Seoane et al., 2002), predicting species responses to changing conditions (e.g., Garcia et al., 2013), and ultimately improving our understanding of the processes that underlie ecological patterns (e.g., Fogarty et al., 2017). Therefore, it is important to understand the limitations of resource selection models to avoid drawing inappropriately generalized conclusions. Because resource selection studies are typically conducted at one study site, and because sites (even within the same region) can differ in the amount, arrangement, and scaling of cover types, we set out to determine whether site location substantially influences the results of a resource selection analysis for a generalist species. Our study demonstrates that—even for one of the most intensively studied species in the world—we may oversimplify resource selection by using a single study site approach. That is, we determined that the importance of different cover types to northern bobwhite is site‐dependent, even for proximal study sites.

Previous studies investigating bobwhite resource selection have revealed a range of results and found little evidence of a single “ideal landscape” for the species (Guthery, 1999). For example, bobwhite can select for bare ground (Lusk et al., 2006) or avoid it (Duquette et al., 2019; Tanner et al., 2016). Similarly, we found there is no universally optimal percent cover of trees or shrubs on a landscape because results are dependent on the structure, availability, and arrangement of woody cover. Though the study sites are close to one another in the context of the bird's entire range, there are marked differences in the site‐level woody cover composition and management practices, which may be driving the differential responses in results. We found a strong selection for shrubs at Beaver River, but no response to tree cover at Packsaddle. The exact mechanism behind the site‐dependent woody cover selection pattern remains speculative and could be related to a number of differences between the two sites. First, Packsaddle undergoes significantly more prescribed fire than Beaver River. Though fire has been shown to have little effect on bobwhite space use (Carroll, Davis, et al., 2017) or density (Ransom et al., 2008), fire likely changes the vegetative functional groups perceived by the Rangeland Analysis Platform, which could partially confound the relationship between bobwhite and shrubs on Packsaddle. In other words, if a shrub‐dominated area used by bobwhite is burned, the Rangeland Analysis Platform may show an increase in herbaceous cover and a concomitant decrease in shrub cover, yet bobwhite are likely to remain in the area (possibly resulting in the neutral relationship between bobwhite and shrubs that we found at Packsaddle). The relationship between bobwhite and shrub cover is more straightforward at Beaver River, where fire is rare, shrubs are more diverse, and bobwhite strongly select for them. This positive association between bobwhite and shrubs at Beaver River is unsurprising because shrubs are a critical bobwhite habitat component (Carroll et al., 2015; Wiseman & Lewis, 1981). Finally, we suspect the strong selection against trees at Packsaddle with no response at Beaver River, to be mostly a function of differences in overall tree cover between the two sites. Because bobwhite tend to have decreased survival in closed‐canopy areas (Howell et al., 2021; Seckinger et al., 2008), it follows that birds may respond differently to trees at Packsaddle, where trees are more abundant, than they would at Beaver River, where trees are an anomaly on the landscape.

Our results indicate there is no universally correct scale of resource selection for bobwhite. This is in alignment with an extensive body of literature underlining the importance of multiscale resource selection (Bauder et al., 2018; Mayor et al., 2009; McGarigal et al., 2016; Timm et al., 2016). Specifically, we found evidence that the scale at which bobwhite select their habitat depends on the habitat feature in focus. Specifically, bobwhite select for shrub cover at intermediate spatial scales (65.61 ha) but they select against tree cover at larger spatial scales (590 ha). The importance of considering scale before drawing conclusions from resource selection studies is well documented in the literature (Bowyer & Kie, 2006; Mayor et al., 2009; McGarigal et al., 2016). For example, mule deer (*Odocoileus hemionus*) in California, USA, were found to select (and avoid) different habitat components at different scales (Kie et al., 2002); however, an unexpected scale—much larger than the deer's home range—was found to be the most informative in predicting deer use and ultimately led to the conclusion that heterogeneity is important for deer conservation. Had management recommendations been developed based on any of the smaller scales, inferior habitat may have been promoted, leading to ineffective management strategies for the species.

Though not a central objective of this study, an interesting finding was that correlated scales are not necessarily perceived equivalently by a species. In other words, even though the woody cover variables we investigated were correlated across spatial scales, there was still a preferred scale in terms of bobwhite resource selection for each cover type (i.e., only one competitive model in our set for both environmental variables). This was unexpected because perfectly correlated scales will always yield identical results, so it stands to reason highly correlated scales will yield highly similar results (i.e., many, or no, competitive models). According to Wiens (1989), ecological phenomena occur along portions of the scale spectrum (spatial grain ranked from small to large), such that they are scale‐independent within their scale domain (i.e., the portion of the scale spectrum where processes are similar enough that generalizations are appropriate). We found bobwhite still showed affinities for some spatial scales over others, regardless of high correlations across habitat variable scales, suggesting that ecological domain boundaries may not be detected by the correlation between scaled environmental data. This finding contributes to a more comprehensive understanding of the role of spatial scale in resource selection studies, which is important because scale is the central factor that determines all observed patterns in ecology (Levin, 1992; Wiens, 1989).

Our study suggests using a single study site approach to examine resource selection is unlikely to extrapolate perfectly across a species' distribution—or even across similar sites. Beaver River and Packsaddle are located on the western periphery of northern bobwhite distribution and, although they have differences in habitat composition, both landscapes are in the same ecoregion with similar broad‐scale habitat (prairie/grassland). Despite these similarities, we found considerable differences in bobwhite resource selection, highlighting the importance of using caution when using single‐site studies to describe resource selection patterns across a species' distribution. However, the difference in resource selection between sites is only one piece of the many sources of variation inherent in ecological systems. Differential selection responses can be found depending on the season (Beck et al., 2013), time of day (e.g., Richter et al., 2020), scale of habitat feature (Mayor et al., 2009), and simply between unique individuals (e.g., Leclerc et al., 2016). One of the central challenges of ecology is managing the trade‐offs between drawing generalized conclusions and maintaining true complexities inherent in nature (Johnson & Lidström, 2018). Balancing practical, generalized conclusions that are easy to implement with precision (more accurate conclusions, but complex and difficult to apply), has presented challenges across ecological concepts including alien species invasions (Johnson & Lidström, 2018), defining species (e.g., Hey et al., 2003), and biological conservation in general (e.g., Lewison et al., 2018). While we acknowledge that it is expensive and inefficient to directly study every area we plan to manage, we caution against setting recommendations for broad extents based on information gathered at small extents. Before extrapolating information beyond the data collected, managers should account for local differences in the availability, arrangement, quality, and scaling of resources. Because large areas encompass higher variability (Fuhlendorf & Smeins, 1996; Wiens, 1989), we recommend managing for large and variable tracts of land that are resilient toward uncertainty.

## CONFLICT OF INTEREST

None declared.

## AUTHOR CONTRIBUTIONS

**Samantha Cady:** Conceptualization (equal); data curation (lead); formal analysis (lead); investigation (lead); methodology (equal); writing–original draft (lead); writing–review and editing (equal). **Craig Davis:** Conceptualization (equal); funding acquisition (equal); investigation (equal); methodology (equal); writing–review and editing (equal). **Samuel D. Fuhlendorf:** Conceptualization (equal); funding acquisition (equal); investigation (equal); methodology (equal); writing–review and editing (equal). **Rheinhardt Scholtz:** Formal analysis (equal); investigation (equal); methodology (equal); writing–review and editing (equal). **Daniel Uden:** Formal analysis (equal); investigation (equal); methodology (equal); writing–review and editing (equal). **Dirac Twidwell:** Investigation (equal); writing–review and editing (equal).

## Data Availability

Northern bobwhite telemetry data from Packsaddle and Beaver River are published to Dryad Digital Repository (https://doi.org/10.5061/dryad.z612jm6cg).

## APPENDIX 1

Distribution of bobwhite trapping effort at Packsaddle and Beaver River from 2012 to 2016. Points represent locations where traps were set, not necessarily where birds were caught.

## APPENDIX 2

Pearson's coefficients indicating correlations across spatial scales for three cover types.

**Table:**

Tree cover					
	7.29 ha	65.61 ha	590 ha	1,149 ha	1,739 ha
0.81 ha	0.918	0.798	0.695	0.668	0.657
7.29 ha		0.912	0.799	0.768	0.754
65.61 ha			0.916	0.882	0.867
590 ha				0.988	0.976
1,149 ha					0.996
**Shrub cover**					
	**7.29 ha**	**65.61 ha**	**590 ha**	**1,149 ha**	**1,739 ha**
0.81 ha	0.934	0.845	0.762	0.741	0.726
7.29 ha		0.936	0.845	0.822	0.805
65.61 ha			0.936	0.910	0.893
590 ha				0.990	0.976
1,149 ha					0.995

## APPENDIX 3

Probability of bobwhite habitat use at Packsaddle and Beaver River WMA as a function of percent tree cover and shrub cover—each across 6 spatial scales.

## APPENDIX 4

Mean percent cover and between‐cell variance of percent cover of 3 cover classes within randomly sampled landscapes across 100 spatial grains. Each blue line is a randomly sampled, 10 km by 10 km landscape in the south‐central semi‐arid prairies.
